# Phenotypic Studies of Natural Killer Cell Subsets in Human Transporter Associated with Antigen Processing Deficiency

**DOI:** 10.1371/journal.pone.0001033

**Published:** 2007-10-17

**Authors:** Jacques Zimmer, Huguette Bausinger, Emmanuel Andrès, Lionel Donato, Daniel Hanau, François Hentges, Alessandro Moretta, Henri de la Salle

**Affiliations:** 1 Laboratoire d'Immunogénétique-Allergologie, Centre de Recherche Public de la Santé (CRP-Santé), Luxembourg City, Luxembourg; 2 INSERM U725, Strasbourg, France; 3 Université Louis Pasteur, Strasbourg, France; 4 Etablissement Français du Sang–Alsace, Strasbourg, France; 5 Service de Médecine Interne, Clinique Médicale B, Hôpitaux Universitaires de Strasbourg, Strasbourg, France; 6 Service de Pédiatrie, Hôpitaux Universitaires de Strasbourg, Strasbourg, France; 7 Dipartimento di Medicina Sperimentale, Sezione di Istologia, Università degli Studi di Genova, Genova, Italy; The Rockefeller University, United States of America

## Abstract

Peripheral blood natural killer (NK) cells from patients with transporter associated with antigen processing (TAP) deficiency are hyporesponsive. The mechanism of this defect is unknown, but the phenotype of TAP-deficient NK cells is almost normal. However, we noticed a high percentage of CD56^bright^ cells among total NK cells from two patients. We further investigated TAP-deficient NK cells in these patients and compared them to NK cells from two other TAP-deficient patients with no clinical symptoms and to individuals with chronic inflammatory diseases other than TAP deficiency (chronic lung diseases or vasculitis). Peripheral blood mononuclear cells isolated from venous blood were stained with fluorochrome-conjugated antibodies and the phenotype of NK cells was analyzed by flow cytometry. In addition, ^51^Chromium release assays were performed to assess the cytotoxic activity of NK cells. In the symptomatic patients, CD56^bright^ NK cells represented 28% and 45%, respectively, of all NK cells (higher than in healthy donors). The patients also displayed a higher percentage of CD56^dim^CD16^−^ NK cells than controls. Interestingly, this unusual NK cell subtype distribution was not found in the two asymptomatic TAP-deficient cases, but was instead present in several of the other patients. Over-expression of the inhibitory receptor CD94/NKG2A by TAP-deficient NK cells was confirmed and extended to the inhibitory receptor ILT2 (CD85j). These inhibitory receptors were not involved in regulating the cytotoxicity of TAP-deficient NK cells. We conclude that expansion of the CD56^bright^ NK cell subtype in peripheral blood is not a hallmark of TAP deficiency, but can be found in other diseases as well. This might reflect a reaction of the immune system to pathologic conditions. It could be interesting to investigate the relative distribution of NK cell subsets in various respiratory and autoimmune diseases.

## Introduction

Human natural killer (NK) cells are phenotypically defined as CD3^−^CD56^+^ lymphocytes. They constitute a heterogeneous blood cell population and subsets can be defined depending on their level of expression of CD16 and CD56. In healthy individuals, CD56^dim^CD16^bright^ cells represent at least 90% of all NK cells [1]. Most of the other NK cells belong to the CD56^bright^ subset that can be further subdivided in a CD16^−^ (30–50% of CD56^bright^) and a CD16^dim^ (50–70% of CD56^bright^) fraction [1]. Finally, minor subpopulations with a CD56^dim^CD16^−^ and conversely CD56^−^CD16^+^ phenotype have been described [2].

The major functional properties of NK cells are cytotoxicity and cytokine production, which are governed by a balance between activating messages transmitted by activating receptors (AR) and inhibitory signals transmitted by inhibitory receptors (IR), respectively [3]–[5]. Activating receptors recognize ligands expressed by tumor cells or stressed cells or, in the case of CD16, antibodies (Ab) of the IgG class bound to target cells. Among the IR, those specific for human leukocyte antigen (HLA) class I molecules recognize either (i) restricted numbers of classical HLA class I alleles in the case of killer immunoglobulin receptors (KIR), (ii) a broad panel of classical HLA class I molecules as well as HLA-G in the case of immunoglobulin-like transcript 2 (ILT2 or CD85j) and (iii) the non classical major histocompatibility complex (MHC) class I molecule HLA-E presenting peptides derived from the signal sequence of classical HLA class I molecules in the case of CD94/NKG2A [4], [5]. In addition, NK cells express also several IR with ligands different from HLA class I molecules [6].

HLA class I molecules present endogenous peptides to cytotoxic CD8^+^ T cells. Newly synthesized HLA class I molecules acquire their peptidic ligands in the endoplasmic reticulum (ER) [7]–[9]. Most of these peptides that result from the degradation of endogenous proteins by the proteasome are translocated into the lumen of the ER by the transporter associated with antigen processing (TAP). TAP is expressed as a heterodimer of two subunits, TAP1 and TAP2, and is inserted in the membrane of the ER. In the absence of a functional TAP, only a very limited amount of peptides can reach the lumen of the ER and associate with HLA class I molecules. Consequently, most of these molecules remain unstable and never reach the cell surface [7]–[9].

Nineteen human cases of TAP deficiency have to date been described [10]–[23]. Clinically, this recessive autosomal disease is very heterogeneous, the spectrum of possible manifestations extending from the complete absence of symptoms [11], [21] to life-threatening conditions [11], [18]. Most frequently, the patients suffer from chronic bacterial infections of the upper and lower airways, evolving to bronchiectasis, and in approximately half of the cases also from skin ulcers with features of a chronic granulomatous inflammation [10], [11]. Recent observations also describe the occurrence of ocular and pulmonary toxoplasmosis [22], [23]. Surprisingly however, severe viral infections have never been reported in these patients, despite the defect in HLA class I-mediated presentation of viral antigens to cytotoxic T cells. It is assumed that other antiviral defense mechanisms like antibodies, non HLA class I-restricted cytotoxic effector cells (γδ T lymphocytes, NK cells) and CD8^+^ T cell responses to TAP-independent antigens are sufficiently efficient [10], [11].

The symptomatic patients have a very large (30- to 100-fold) reduction in the cell surface expression levels of HLA class I molecules, as compared to healthy individuals [10], [11]. As NK cells preferentially kill target cells with low or absent HLA class I molecules on the surface [4], we previously investigated susceptibility of autologous cells to NK cell-mediated lysis in two TAP-deficient patients. TAP-deficient cells are killed by TAP^+^ normal NK cells but not by autologous resting NK cells. Nevertheless, resting TAP^−^ NK cells are able to perform antibody-dependent cellular cytotoxicity (ADCC) to some extent, and after stimulation with interleukin (IL) 2, they become cytotoxic not only towards tumor cell lines, but also to two types of autologous cells, namely Epstein-Barr virus (EBV)-transformed B lymphoblasts [24] and skin fibroblasts [25]. Their repertoire of AR and IR is almost normal, although CD94/NKG2A is expressed at much higher levels than by normal NK cells [24]. Not only AR, but also IR are functional [24], [26], as assessed using activated TAP^−^ NK cells in cytotoxicity tests against allogeneic normal cells. Thus, the mechanism responsible for the hyporesponsiveness of resting TAP-deficient NK cells is still unknown.

Previous investigations also revealed a higher than normal percentage of CD56^bright^ cells among the patients' NK cells [26]. The objective of our study was to investigate if the high percentage of CD56^bright^ NK cells and the over-expression of NKG2A were constant findings in TAP deficiency, and if these features could also be observed in diseases of other origins. Therefore, we compared by flow cytometry peripheral blood NK cells from the symptomatic TAP-deficient patients to those from two asymptomatic patients [21] as well as to a small panel of individuals with respiratory diseases of etiologies different from TAP deficiency and of vasculitis patients. A group of normal control donors was also included.

## Methods

### Participants

Blood samples were obtained from volunteers or patients attending the clinics of Strasbourg University Hospitals and were collected during routine clinical (diagnostic/prognostic/therapeutic) procedures prescribed by two co-authors, Pr. E. Andrès and Dr. L. Donato. Before blood samples were collected for the study, all healthy donors, all patients and, for those patients who were minor at the time of blood sampling, their parents, gave written informed consent in agreement with the Helsinki Declaration and French legislation, under which no approval by an Ethics Committee was required in this case.

We included four patients with a known TAP deficiency [12], [21], two patients with a known cystic fibrosis, five patients with chronic respiratory diseases complicated by acute infections, five patients with chronic respiratory diseases but no acute infection, and five vasculitis patients. The inclusion in one or another group was decided on the basis of clinical and biological parameters by the above mentioned physicians. As our study was intended as preliminary and as potential indicator for the interest of future large scale investigations, we deliberately limited the size of the different groups, and no sample size calculations were performed. In addition, we included seventeen volunteer donors (the same number as patients with no TAP deficiency) with no apparent signs of disease.

### Flow cytometry

Peripheral blood mononuclear cells (PBMC) were isolated from citrated venous blood by Ficoll-Hypaque density gradient centrifugation and either used directly for flow cytometry or frozen in RPMI 1640 culture medium with 20% fetal calf serum (FCS) and 10% dimethylsulfoxyde (DMSO).

For flow cytometry stainings, PBMC were washed at 4°C, once in RPMI 1640/10% FCS and once in phosphate buffered saline (PBS) with 3% FCS (staining buffer). Cells were then resuspended in ice-cold staining buffer at 5×10^6^ cells/ml and stained in aliquots of 100 µl with various purified or fluorochrome-conjugated Ab at optimal concentrations previously determined by titration. The Ab and fluorochromes used are listed in table 1. They were purchased from Beckman Coulter (Margency, France), with the exception of HP-F1 (anti-ILT2), generously provided by Pr. Miguel López-Botet, University Pompeu Fabra, Barcelona, Spain, and Z199 (anti-NKG2A), produced in one of our labs and previously described [27]. After incubation in the dark, at 4°C, for 30 minutes, cells were washed twice in ice-cold staining buffer and fixed in Cytofix solution (BD Biosciences, Erembodegem, Belgium). In the case of cells first stained with purified Ab, they were washed, incubated with a secondary PE-conjugated goat anti-mouse Ab (Dako, Glostrup, Denmark), washed again, incubated in mouse serum for 20 minutes, washed once more, and finally stained with fluorochrome-conjugated Ab as described above. Stained cells were kept in the dark at 4°C until flow cytometry, that was performed on a FACSCalibur™ flow cytometer (BD Biosciences). Results were analyzed with Cellquest™ software (BD Biosciences). The region of living lymphocytes was gated based on forward and side scatter characteristics, and at least 2500 NK cells per sample were acquired.

**Table 1:** Antibodies used for flow cytometry

antibody (clone name)	fluorochrome	antigen	supplier
T11	FITC	CD2	Beckman Coulter
UCHT1	FITC or PC5	CD3	Beckman Coulter
3G8	FITC	CD16	Beckman Coulter
J4.119	FITC or PC5	CD19	Beckman Coulter
N901 (NKH-1)	PE or PC5	CD56	Beckman Coulter
HP-F1	PE*	CD85j (ILT2)	Pr. M. López-Botet
Z199	PE*	CD159 (NKG2A)	Pr. A. Moretta [27]

FITC : fluorescein isothiocyanate ; PE : phycoerythrin ; PC5 : phycoerythrin-cyanin 5. *: indirect staining; cells were first stained with the indicated purified Ab, washed, and then stained with a secondary goat anti-mouse Ab conjugated to PE. All other Ab were used in directly fluorochrome-conjugated forms.

### Cytotoxicity Assays

Activated NK cells and phytohemagglutinin (PHA)-induced T cell blasts were obtained in culture as previously described [26]. For the assessment of NK cell cytotoxicity, standard four hour ^51^Chromium release assays were performed as previously described [24], [26] in the absence or presence of masking Ab used at a concentration of 10 µg/ml.

## Results

### High percentage of CD56^bright^ NK cells in some patients with TAP deficiency but also chronic diseases of other origins

**Figure 1:**
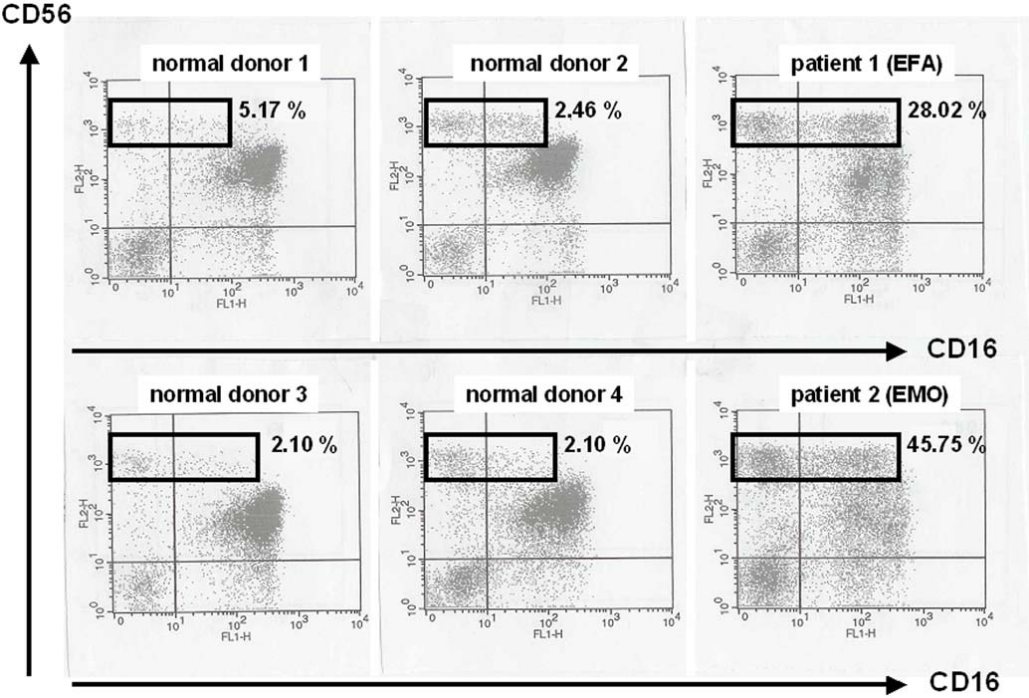
High percentages of CD56^bright^ NK cells in two symptomatic TAP-deficient patients. PBMC from four normal donors and the patients were stained with fluorochrome-conjugated antibodies and analyzed by flow cytometry. Expression of CD16 and CD56 is shown on gated CD3^−^CD19^−^ lymphocytes (one representative experiment). Percentages of CD56^bright^ NK cells among total NK cells are indicated for each donor.

To confirm and extend our previous findings about the high percentage of CD56^bright^ NK cells in two TAP-deficient patients [26], we stained PBMC from these individuals and from seven healthy donors with anti-CD3, -CD19, -CD16 and -CD56 Ab. Within the gate of CD3^−^CD19^−^ lymphocytes corresponding to NK cells, we analyzed CD16 *versus* CD56 expression. As shown in figure 1 and in table 2, it appeared indeed that the two patients had a very high percentage of CD56^bright^ NK cells compared to normal donors. CD56^bright^ NK cells were distributed quite equally between the CD16^−^ and CD16^dim^ subsets in patient EMO, whereas the CD16^dim^ population was predominant in patient EFA. In addition, both patients also had a higher percentage of CD56^dim^CD16^−^ NK cells (table 2).

**Table 2:** NK cell subsets in TAP-deficient patients and healthy control donors

	CD56^bright^ CD16^−^	CD56^bright^ CD16^dim^	Total CD56^bright^	CD56^dim^ CD16^bright^	CD56^dim^ CD16^−^
**HD 1**	3.51	1.66	5.17	91.38	3.45
**HD 2**	2.12	0.34	2.46	96.27	1.27
**HD 3**	1.35	0.75	2.10	97.20	0.70
**HD 4**	1.35	0.75	2.10	94.70	3.20
**HD 5**	4.18	4.29	8.47	91.36	0.17
**HD 6**	4.57	3.24	7.81	92.14	0.05
**EHA (HD 7)**	3.55	2.98	6.53	92.05	1.42
**EFA**	8.56	19.46	28.02	52.82	19.16
**EMO**	21.93	23.82	45.75	42.11	12.14
**SFH**	5.16	3.19	8.35	86.37	5.28
**DFH**	2.65	2.08	4.73	92.04	3.23

Values correspond to the cells in the different subsets expressed as percentages of total peripheral blood NK cells. HD: healthy donor; EHA: father of patients EFA and EMO; EFA, EMO: symptomatic TAP2-deficient patients (siblings); SFH, DFH: asymptomatic TAP2-deficient patients (siblings).

As CD56^bright^ NK cells are less cytotoxic than CD56^dim^ NK cells [1], [28], their high percentage in TAP-deficient patients might reflect an adaptation to the low HLA class I expression in order to avoid cytotoxicity toward autologous targets. In that case, a similar distribution of NK cell subsets should be observed in other TAP-deficient individuals. Therefore, we looked at PBMC from two additional patients, SFH and DFH [21], and found that their CD56^bright^ NK cells represented 8% and 4% of all peripheral blood NK cells, respectively (table 2). They were thus within the normal range (<10% of NK cells), as were the CD56^dim^CD16^−^ cells.

In the absence of a systematic expansion of the CD56^bright^ NK cell fraction in TAP-deficient patients, an alternative explanation for the values observed in patients EFA and EMO could be that CD56^bright^ NK cells become more abundant as a reaction to an acute infection or to the chronic inflammatory state present in these patients (whereas patients SFH and DFH were asymptomatic). Should this be true, higher than normal percentages of CD56^bright^ NK cells might also be found in patients with no TAP deficiency but with acute or chronic infections and/or inflammatory diseases of other origins.

We thus repeated the stainings described above in a small series of patients mentioned in the Methods section. Details about these patients are listed in table 3. HLA class I deficiency was ruled out in all of them by flow cytometry staining of PBMC with the pan anti-HLA class I Ab W6/32 (data not shown). We also included a panel of healthy donors. The results summarized in table 4 revealed, in accordance with our hypothesis, a percentage of the CD56^bright^ population clearly above 10% of all peripheral blood NK cells in five of the seventeen patients (29.41%) and even in three of the ten healthy donors (30%). Regarding the CD56^dim^CD16^−^ NK cell subtype, it was higher than 10% in four patients (23.53%), of whom only one also displayed a high percentage of CD56^bright^ NK cells in parallel (table 4).

**Table 3:** Characteristics of patients with diseases other than TAP deficiency

patient number	gender	age*	diagnosis
1	F	5	cystic fibrosis
2	F	14	cystic fibrosis
3	M	71	chronic respiratory insufficiency, acute infection
4	M	72	COPD, acute infection
5	M	43	bronchiectasis, acute infection
6	M	59	chronic respiratory insufficiency, acute infection
7	F	88	chronic respiratory insufficiency, acute infection
8	M	86	COPD, no acute infection
9	M	74	COPD, no acute infection
10	F	81	chronic respiratory insufficiency, pneumoconiosis
11	F	72	chronic respiratory insufficiency
12	M	71	chronic respiratory insufficiency
13	F	92	cryoglobulinemia, leucocytoclastic vasculitis
14	F	27	vasculitis
15	F	88	suspected vasculitis
16	F	43	vasculitis
17	F	76	vasculitis

age*: age at the time of blood sampling; F: female; M: male; COPD: chronic obstructive pulmonary disease.

**Table 4:** NK cell subsets in additional healthy donors and patients with diseases other than TAP deficiency

	CD56^bright^ CD16^−^	CD56^bright^ CD16^dim^	Total CD56^bright^	CD56^dim^ CD16^bright^	CD56^dim^ CD16^−^
HD 8	3.63	3.09	6.72	88.94	4.34
HD 9	4.56	3.42	7.98	89.52	2.50
HD 10	5.78	3.30	9.08	88.79	2.13
HD 11	11.43	5.54	16.97	80.00	3.03
HD 12	11.93	3.03	14.96	79.41	5.63
HD 13	8.56	4.60	13.16	82.38	4.46
HD 14	3.60	3.24	6.84	91.04	2.12
HD 15	2.66	3.75	6.41	92.24	1.25
HD 16	3.20	1.71	4.91	85.34	9.75
HD17	3.08	0.39	3.47	95.76	0.77
1	7.45	5.54	12.99	85.25	1.76
2	3.91	0.00	3.91	83.59	12.50
3	2.97	1.16	4.07	91.75	4.18
4	1.76	1.23	2.99	94.25	2.76
5	8.30	4.30	12.60	81.88	5.52
6	6.74	18.19	24.95	68.59	6.46
7	2.51	1.11	3.62	86.77	9.61
8	4.67	1.56	6.23	90.00	3.77
9	2.23	0.49	2.72	92.27	5.01
10	1.65	0.78	2.43	95.58	1.99
11	4.99	1.78	6.77	85.24	7.99
12	3.42	1.57	4.99	77.62	17.39
13	9.09	1.08	10.17	83.77	6.06
14	5.43	2.59	8.02	78.12	13.86
15	5.37	10.40	15.77	83.70	0.53
16	5.85	4.35	10.20	88.11	1.69
17	20.69	4.83	25.52	40.00	34.48

HD 8–HD 17: healthy donors (different from those shown in table 2); 1–17: patients 1–17 with diseases other than TAP deficiency (see table 3 for details).

### Over-expression of NKG2A on both CD56^bright^ and CD56^dim^ NK cells from two TAP-deficient patients

**Figure 2:**
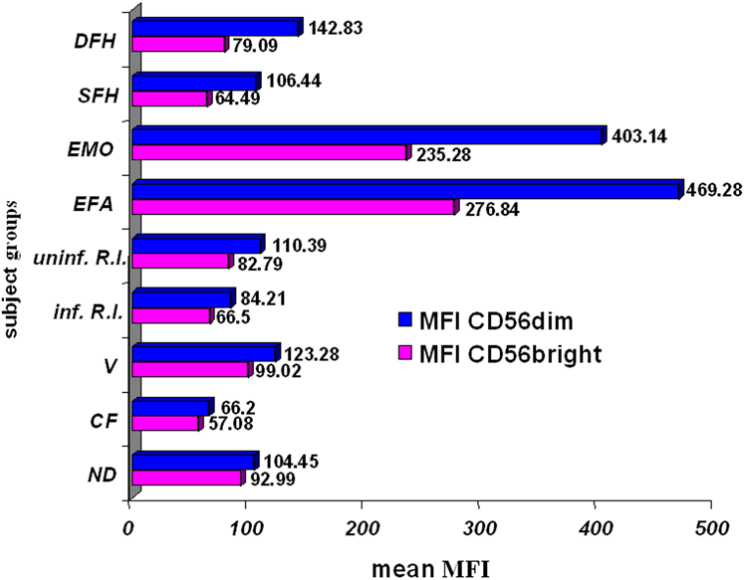
Dramatic over-expression of NKG2A on both CD56^bright^ and CD56^dim^ NK cell subsets of symptomatic TAP-deficient patients. Mean fluorescence intensities (MFI) of NKG2A were expressed relative to the values of a randomly chosen healthy donor arbitrarily considered as 100%. Mean values of different subject groups were calculated. ND: normal donors (n = 17); CF: cystic fibrosis patients (N = 2), V: vasculitis patients (n = 5), inf. R.I.: infected respiratory insufficiency (n = 5), uninf. R.I.: uninfected respiratory insufficiency (n = 5); EFA, EMO: symptomatic TAP-deficient patients; DFH, SFH: asymptomatic TAP-deficient patients.

The previously reported over-expression of the IR CD94/NKG2A by NK cells from patients EFA and EMO [24] could likewise be interpreted as an adaptation of NK cells to the low levels of HLA class I molecules in these patients, because it might allow to avoid autoreactivity, at least to some extent. However, as it is known that mean fluorescence intensities (MFI) of NKG2A are much higher on CD56^bright^ than on CD56^dim^ NK cells [1], [28], the observed over-expression of NKG2A in EFA and EMO might simply be a consequence of the high percentage of CD56^bright^ NK cells and would not necessarily reflect a regulatory mechanism. To address this point, we stained PBMC from EFA and EMO, SFH, DFH, the seventeen healthy donors and the seventeen patients with diseases other than TAP deficiency with anti-CD3, -CD19, -CD56 and -NKG2A Ab and analyzed NKG2A expression in terms of percentages of positive cells and MFI on gated CD3^−^CD19^−^CD56^+^ NK cells. Among CD56^bright^ NK cells, more than 90% expressed NKG2A in the vast majority of healthy donors as well as patients (mean: 90.75%, range: 56–100%). Much more inter-individual differences were observed for CD56^dim^ NK cells, of whom a mean of 49.37% were NKG2A^+^ (range: 20–77%). To compare MFI, we randomly designed a healthy donor whose values of NKG2A MFI on both NK cell subsets were arbitrarily considered to be 100%. The values measured in all the other control donors and in the patients were then expressed relative to this reference value, and we calculated means of MFI for the different subject groups (healthy donors and patients, the latter grouped according to similar disease states). Interestingly, it appeared that NK cells from EFA and EMO, but not from SFH and DFH, dramatically over-expressed NKG2A on both CD56^bright^ and CD56^dim^ NK cells (figure 2). The difference to the other subject groups was even more pronounced for CD56^dim^ than for CD56^bright^ NK cells. Mean values of healthy donors and other patients were much lower (figure 2). When looking at every individual, none of the healthy donors and the other patients, including those with increased CD56^bright^ percentages, displayed such high MFI of NKG2A on either subset (data not shown). In accordance with the literature [1], [28], the higher expression level of NKG2A on CD56^bright^, compared to CD56^dim^ NK cells, was clearly confirmed in each individual except EFA and EMO with comparable MFI on both subsets (data not shown). Thus, in contrast to the proportion of CD56^bright^ NK cells that might increase in several different pathologic conditions, over-expression of NKG2A was found exclusively in the two symptomatic TAP-deficient patients.

### The inhibitory receptor ILT2 is over-expressed in symptomatic and asymptomatic TAP-deficient patients

**Figure 3:**
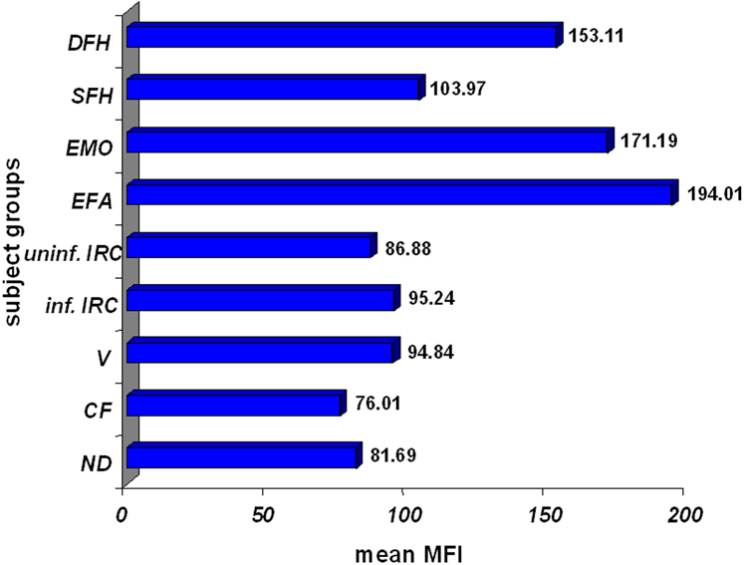
Over-expression of ILT2 on CD56^dim^ NK cells of symptomatic and asymptomatic patients. Mean fluorescence intensities (MFI) of ILT2 were expressed relative to the values of a randomly chosen control donor arbitrarily considered as 100%. Mean values of different subject groups were calculated. ND: normal donors (n = 17); CF: cystic fibrosis patients (N = 2), V: vasculitis patients (n = 5), inf. R.I.: infected respiratory insufficiency (n = 5), uninf. R.I.: uninfected respiratory insufficiency (n = 5); EFA, EMO: symptomatic TAP-deficient patients; DFH, SFH: asymptomatic TAP-deficient patients.

We asked if the broad spectrum IR ILT2 might likewise be over-expressed at the surface of TAP-deficient NK cells, whereas we had previously shown that members of the KIR family, with a restricted number of HLA class I ligands, are normally expressed in these patients [24]. As previously reported by others [1], [28], ILT2 was predominantly, but not exclusively, expressed by CD56^dim^ NK cells, and percentages of ILT2^+^ NK cells considerably differed between donors. Regarding the expression levels of ILT2, that we considered only on CD56^dim^ NK cells, they were strongly augmented on CD56^dim^ NK cells from EFA, EMO and DFH, although not as importantly as for NKG2A. SFH NK cells also over-expressed ILT2 to some extent compared to the mean of MFI from healthy donors and patients other than those with TAP deficiency (figure 3). Mean values of different subject groups were calculated in the same way as for NKG2A (see above). At the individual level, one control donor and five patients had MFI higher than SFH but clearly lower than the three other TAP-deficient patients. Interestingly, MFI of ILT2 on T cells, B cells and monocytes from TAP-deficient patients were not increased but were in the range of the values seen in the healthy donors and the other patients (data not shown), which suggests that the over-expression of ILT2 specifically by NK cells might have some biological significance. Even if care should be taken in the context of the low number of TAP-deficient individuals included in this study, our results nevertheless indicate that ILT2 over-expression might be a more consistent finding in this disease than NKG2A over-expression, and that it might be less dependent on the presence of clinical symptoms.

### Lysis of TAP-deficient PHA-induced T cell blasts is not restored by antibodies masking autologous NK cell inhibitory receptors

**Figure 4:**
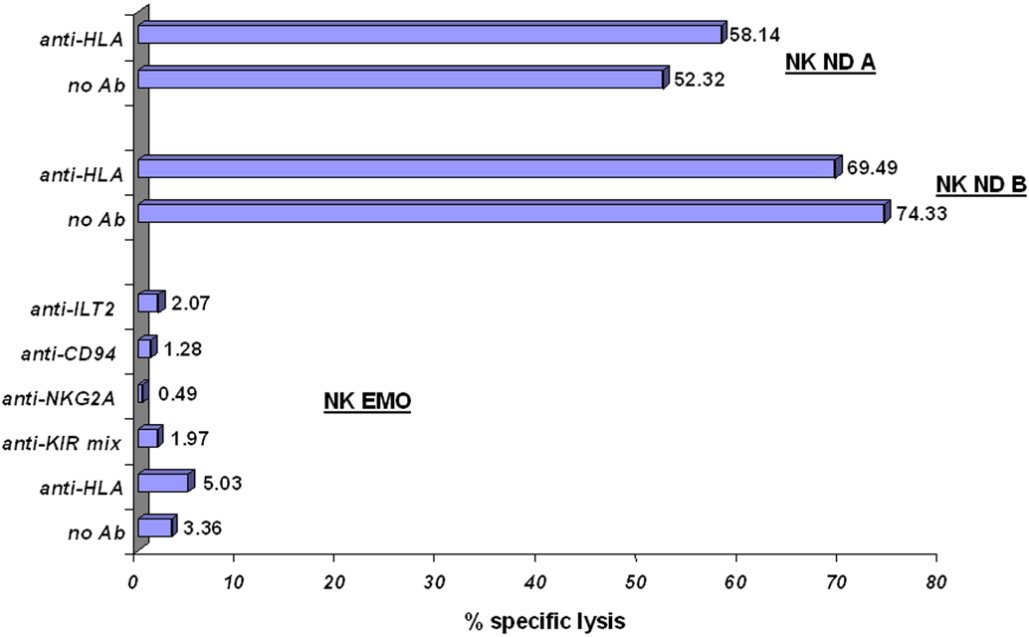
TAP-deficient PHA-induced T cell blasts are resistant to killing by activated autologous NK cells even in the presence of antibodies masking inhibitory receptors. A standard ^51^Chromium release assay was performed with activated NK cells from two normal donors (NK ND A, NK ND B) and of patient EMO (NK EMO) as effectors and TAP-deficient PHA-induced T cell blasts from patient EMO as targets. The effector to target (E/T) ratio was 6/1. Masking Ab were used at a concentration of 10 µg/ml. Indicated values correspond to the percentages of specific lysis.

We previously demonstrated [24], [25] that activated NK cells from TAP-deficient patients strongly lyse autologous EBV-transformed B lymphoblasts and skin fibroblasts. NK cell inhibitory receptors are therefore unlikely to play any protective role in this situation. In contrast, PHA-induced T cell blasts of the patients are resistant to autologous NK cell-mediated killing, whereas masking of HLA class I molecules with Ab does not restore lysis [26]. To complete these studies, we performed cytotoxicity assays with activated NK cells from patient EMO as effectors and autologous PHA-induced T cell blasts as targets, in the absence or presence of several Ab. Confirming our previous results [26], target cells were completely protected from lysis in the absence of Ab and in the presence of anti-HLA Ab (figure 4). Interestingly, killing did likewise not appear in the presence of masking Ab against KIR, ILT2 and CD94/NKG2A (figure 4), which suggests that HLA class I-specific IR, even the over-expressed ones, do not play a role in the protection of PHA-induced T cell blasts from autologous NK cell-mediated lysis. The results cannot be explained by a general resistance of PHA-induced T cell blasts from patient EMO to NK cell-mediated killing, because they were highly susceptible to activated NK cells from two normal unrelated donors (figure 4).

### Progressive reduction of the CD56^bright^ NK cell fraction with improved clinical status of TAP-deficient patients

The results shown for patients EFA and EMO were obtained with PBMC harvested at the time when their TAP deficiency was diagnosed and their respiratory symptoms were very severe. Clinical status progressively improved over the following years due to an intense therapeutic approach with repeated intravenous administration of antibiotics and chest physiotherapy. In parallel, we observed a reduction of the percentages of CD56^bright^ NK cells in both patients, even if the values measured remained substantially higher than in healthy donors. Interestingly, CD56^bright^CD16^−^ proportions remained globally unchanged, whereas the lower percentages of CD56^bright^CD16^dim^ NK cells accounted for most of the lowering. Over time, expression levels of NKG2A strongly diminished in both NK cell subsets of patient EFA (yet remained higher than in normal NK cells), and only moderately in patient EMO. Reductions of MFI of ILT2 were modest. The percentage of CD56^dim^CD16^−^ NK cells strongly decreased with time in patient EFA, but slightly increased in patient EMO. At least regarding CD56^bright^ NK cells, the reduced percentage that parallels clinical improvement could be in favor of a link between the proportion of this subset among total NK cells and the intensity and severity of respiratory infections.

## Discussion

In this paper, we confirm and extend our previous findings [26] that in two TAP2-deficient patients, the percentage of CD56^bright^ NK cells among total peripheral blood NK cells is very high in comparison to healthy donors. To our knowledge, NK cell subset distribution has not yet been investigated in detail in TAP-deficient patients studied by other groups, so that it is currently unknown if the distribution of CD56^bright^ and CD56^dim^ NK cells is similar or not in these other cases. The two asymptomatic TAP-deficient individuals [21] display anyhow normal percentages of CD56^bright^ and CD56^dim^ NK cells and thus the increased proportion of CD56^bright^ NK cells is not a constant hallmark of human TAP deficiency.

Based on this finding, we reasoned that the high percentage of CD56^bright^ NK cells could be a consequence of the chronic infectious and inflammatory state of the patients. Indeed, among patients with chronic inflammatory diseases other than TAP deficiency, approximately 30% displayed CD56^bright^ percentages above 10% of total NK cells, although their values never reached those of the TAP-deficient individuals. Due to the small number and the heterogeneity of our series that was intended as part of a preliminary study, we cannot conclude if CD56^bright^ NK cell percentages are preferentially increased in certain well-defined disease stages or subtypes, or if this might rather occur, irrespective of the underlying condition, in response to infections with particular but not all microorganisms. These points would be very interesting to address in future large scale studies, as well as the question if CD56^bright^ NK cell expansions could be used as a prognostic marker for the short- or long-term outcome of some diseases.

We were somehow surprised to observe high CD56^bright^ NK cell percentages also among 30% of the healthy control donors we tested, as it is generally admitted that in this population, CD56^bright^ NK cells do not exceed 10% of all peripheral blood NK cells [1], [29]. Information about acute infectious episodes preliminary to blood sampling is not available from the group of healthy donors. This finding would likewise benefit from being validated by much larger series of individuals, whereby the criteria for attribution of the “healthy donor” status should be carefully defined.

Expansions of CD56^bright^ NK cells are known during reconstitution of the immune system after bone marrow grafts, where the first lymphocytes to reappear in peripheral blood usually are CD56^bright^ NK cells, and these cells also expand in patients daily treated with a low dose of IL-2 [30]. More recently, Cac and Ballas [31] published the case of a female patient with recalcitrant periungual warts whose peripheral blood NK cells contrasted with the usual situation, as the vast majority of her NK cells were of the CD56^bright^ type and only a minor subset was CD56^dim^. In an atypical case of X-SCID with Omenn syndrome-like manifestations, the patient displayed an increased number of peripheral blood NK cells, half of them being of the CD56^bright^ subtype [32].

The latter observations raise the question of the relationship between both peripheral blood NK cell subsets, which is quite a controversial issue still not clearly resolved. The two main views are that either (i) CD56^bright^ NK cells are immature precursors of CD56^dim^ NK cells, or that (ii) both NK cell types are functionally different, terminally differentiated effectors that arise from separate hematopoietic precursors. Cited arguments in favor of the former hypothesis are the small number of CD56^bright^ NK cells in peripheral blood, their proliferation in the presence of picomolar concentrations of IL-2, the expression of the hematopoietic stem cell marker CD117, the low content of cytotoxic granules and the low cytotoxic activity [30], [33], [34]. However, CD56^bright^ and CD56^dim^ NK cells display very different repertoires of adhesion molecules and chemokine receptors [1], [35] and thus different migratory properties. In addition, CD56^bright^ NK cells produce much higher amounts of cytokines than their CD56^dim^ counterparts, so that both subtypes might well be distinct and specialized effector cell populations [1], [35], [36]. In a very recent review paper [37], the Caligiuri group, in contrast to previous data in favor of the distinct effector cell hypothesis [36], present a linear sequential model of human NK cell development, in which CD56^bright^ NK cells would again be precursors of CD56^dim^ NK cells. On the other hand, comparative microarrays between purified CD56^bright^ and CD56^dim^ NK cells are strongly in favor of the hypothesis of two distinct subtypes [38].

The findings of our study do not allow to strengthen one rather than another of these models. If one considers that both NK cell types are distinct effectors, it could be conceivable that CD56^bright^ NK cells are increased during infectious and/or inflammatory processes because the immune system is globally activated and high amounts of NK cell-derived cytokines are needed as part of the immune response. The corresponding NK cell subset would therefore expand in these situations before going back to baseline levels once the stimuli have disappeared. In support of this idea, a child with human herpes virus 6 (HHV-6)-associated acute necrotizing encephalopathy had a very high percentage of CD56^bright^ NK cells in peripheral blood [39], and multiple sclerosis patients treated with IFN-β display a progressive increase in the percentages of CD56^bright^ NK cells [40]. Alternatively, during such disease states, the turnover of CD56^dim^ NK cells might be very high and the number of new NK cells generated from bone marrow precursors would increase through a feedback mechanism. The consequence would then be a high percentage of immature CD56^bright^ NK cell precursors that appear in peripheral blood to continuously replace CD56^dim^ NK cells.

In contrast to the increased percentages of CD56^bright^ NK cells, over-expression of the IR NKG2A and ILT2 was exclusively observed in the TAP-deficient patients. The former molecule was dramatically up-regulated on both CD56^bright^ and CD56^dim^ NK cell subsets of the two symptomatic individuals, whereas ILT2 was over-expressed, although to a lesser extent than NKG2A, by CD56^dim^ NK cells from all four TAP-deficient patients. Interestingly, the over-expression only implicates IR with a broad spectrum of HLA class I ligands, but no receptors of the KIR family [24] that have a more restricted panel of ligands. From a finalistic point of view, this could be interpreted as an adaptation of NK cells to the low HLA class I-expressing environment in which they develop, as a higher expression level of broad-spectrum IR might be able to still transmit enough inhibitory signals to the NK cells and thus contribute to self-tolerance. On the other hand, recent papers [41]–[43] have convincingly shown that NK cells have in fact to express IR for self MHC class I molecules to become “licensed” [41], “armed” [42] or “educated” [43] for functional activity, and that they remain “hyporesponsive” in their absence. Thus, as resting NK cells from TAP-deficient patients express such receptors but have no cytotoxic activity, one might postulate another “licensing mechanism” that would be deficient in these patients, or alternatively that HLA class I levels on surrounding cells would simply be too low to correctly educate NK cells. Further work is clearly needed to clarify these issues. However, it should also be taken into account that even resting TAP-deficient NK cells perform ADCC, and that IL-2-activated NK cells from these patients are cytotoxic towards tumor cell lines and some (EBV-transformed B cells and fibroblasts) but not other (PHA-induced T cell blasts) autologous targets, which suggests that they might have underwent some educational mechanism that partly failed because self-tolerance is not constantly maintained

In the mouse, it has been shown that the inhibitory NK cell receptor Ly49A interacts with its ligands H-2D^d^ or H-2D^k^ through a *cis* interaction [44], which means that receptor and ligand are closely associated in the membrane of a same NK cell. This *cis* interaction has important functional consequences and actually governs the inhibitory potential of Ly49A as well as its accessibility for tetramer and Ab staining [44]. It could thus be argued that NKG2A and/or ILT2 are not truly over-expressed in TAP deficiency, but that the lack of ligands and, consequently, the absence of *cis* interaction would render them simply more accessible for specific Ab. At least for NKG2A, this explanation seems rather unlikely, as (i) over-expression of NKG2A is not a constant finding in TAP deficiency, and (ii) expression levels diminish in parallel to clinical improvement. Regarding ILT2, the possibility still exists but would have to be tested in the context of the crystal structure of this receptor.

Although the over-expression of some IR might be an adaptive mechanism to the low levels of HLA class I expression, it does not seem to have any functional role in the regulation of the cytotoxic activity of IL2-activated TAP-deficient NK cells, as revealed by the absence of killing of autologous PHA-induced T cell blasts even in the presence of anti-IR masking Ab. These data, in accordance with our previous hypothesis [26], are in favor of the existence of an IR with ligands different from HLA class I molecules and that would be strongly expressed by activated TAP-deficient NK cells, the ligands being present on the surface of PHA-induced T cell blasts, but not of EBV-transformed B cells nor skin fibroblasts. With regard to the identity of this IR, additional investigations are required, although Markel et al. [20], who studied another family affected by TAP deficiency, have shown that CD66a or CEACAM1 is over-expressed by NK cells of these patients and functions as an IR protecting autologous cells. However, the patients also had a reduced expression level of the AR NKp46, which is not the case in our patients [26]. Thus, several different adaptive mechanisms of NK cells might exist according to the individual cases.

In conclusion, in this paper, we provide data that extend our knowledge on the phenotype of peripheral blood NK cells from TAP-deficient patients. In addition, we show that percentages of CD56^bright^ NK cells might exceed 10% of all peripheral blood NK cells in a substantial fraction of patients with chronic diseases other than TAP deficiency and even in donors considered as immunologically normal. Therefore, it might be recommendable to check more frequently the NK cell subset distribution in infectious and inflammatory diseases, in order to precise the conditions under which CD56^bright^ NK cells expand *in vivo*, and what is the pathophysiological significance of this phenomenon.
